# Fecal microbiota transplantation ameliorates atherosclerosis in mice with C1q/TNF-related protein 9 genetic deficiency

**DOI:** 10.1038/s12276-022-00728-w

**Published:** 2022-02-03

**Authors:** Eun Sil Kim, Bo Hyun Yoon, Seung Min Lee, Min Choi, Eun Hye Kim, Byong-Wook Lee, Sang-Yeob Kim, Chan-Gi Pack, Young Hoon Sung, In-Jeoung Baek, Chang Hee Jung, Tae-Bum Kim, Jin-Yong Jeong, Chang Hoon Ha

**Affiliations:** 1 Department of Convergence Medicine and Asan Institute for Life Sciences, Asan Medical Center, University of Ulsan College of Medicine, Seoul, Republic of Korea; 2 ConveRgence mEDIcine research cenTer (CREDIT), Asan Institute for Life Sciences, Asan Medical Center, Seoul, Republic of Korea; 3 Department of Internal Medicine, Asan Medical Center, University of Ulsan College of Medicine, Seoul, Republic of Korea; 4 Department of Allergy and Clinical Immunology, Asan Medical Center, University of Ulsan College of Medicine, Seoul, Republic of Korea; 5 Digestive Diseases Research Center, University of Ulsan College of Medicine, Seoul, Republic of Korea

**Keywords:** Atherosclerosis, Experimental models of disease, Mechanisms of disease

## Abstract

Despite the strong influence of the gut microbiota on atherosclerosis, a causal relationship between atherosclerosis pathophysiology and gut microbiota is still unverified. This study was performed to determine the impact of the gut microbiota on the pathogenesis of atherosclerosis caused by genetic deficiency. To elucidate the influence of the gut microbiota on atherosclerosis pathogenesis, an atherosclerosis-prone mouse model (C1q/TNF-related protein 9-knockout (CTRP9-KO) mice) was generated. The gut microbial compositions of CTRP9-KO and WT control mice were compared. Fecal microbiota transplantation (FMT) was performed to confirm the association between gut microbial composition and the progression of atherosclerosis. FMT largely affected the gut microbiota in both CTRP9-KO and WT mice, and all transplanted mice acquired the gut microbiotas of the donor mice. Atherosclerotic lesions in the carotid arteries were decreased in transplanted CTRP9-KO mice compared to CTRP9-KO mice prior to transplantation. Conversely, WT mice transplanted with the gut microbiotas of CTRP9-KO mice showed the opposite effect as that of CTRP9-KO mice transplanted with the gut microbiotas of WT mice. Here, we show that CTRP9 gene deficiency is related to the distribution of the gut microbiota in subjects with atherosclerosis. Transplantation of WT microbiotas into CTRP9-KO mice protected against the progression of atherosclerosis. Conversely, the transplantation of CTRP9-KO microbiotas into WT mice promoted the progression of atherosclerosis. Treating atherosclerosis by restoring gut microbial homeostasis may be an effective therapeutic strategy.

## Atherosclerosis: A role for gut microbes

Transplanting fecal matter into the guts of mice used as a model of atherosclerosis suggests a role for gut microbes in causing, preventing and potentially treating this serious condition. Atherosclerosis is the build-up of fatty deposits in artery walls, often called hardening of the arteries. Eun Sil Kim and colleagues at Asan Medical Center, University of Ulsan College of Medicine in Seoul, South Korea, analyzed the gut microbes of mice that had been genetically altered to develop atherosclerosis. The gut microbial populations of the mice with atherosclerosis were significantly different from those of normal mice used as controls. Fecal transplantation from control mice into atherosclerotic mice halted the progression of atherosclerosis, with transplantation in the opposite direction promoting atherosclerosis. Macrophage cells of the immune system seem to be involved in the protective effect of beneficial gut microbes.

## Introduction

Complex and diverse microbial communities are formed in the human gut to maintain a symbiotic relationship and homeostasis^1,2^. The gut microbiota has emerged as a critical factor in human health and disease^3–5^. Evidence to date suggests that changes in the composition and diversity of the gut flora confer a high risk of developing cardiovascular diseases (CVDs), such as arteriosclerosis, hypertension, heart failure, stroke, atrial fibrillation, and myocardial fibrosis^6,7^.

Atherosclerosis is a chronic inflammatory condition of blood vessels characterized by the infiltration of macrophages, T cells, and other immune cells that, along with cholesterol, form atherosclerotic plaques^8,9^. Atherosclerotic plaques are influenced by blood shear stress and atheroprotective genes in endothelial cells^10^. In a previous study of oral and gut samples from patients with atherosclerosis, the abundance of *Veillonella* and *Streptococcus* in atherosclerotic plaques correlated with their abundance in the oral cavity, suggesting that the plaque microbiota may correlate with disease markers of atherosclerosis^11^. Furthermore, patients with symptomatic atherosclerosis had a higher relative abundances of *Anaeroglobus* in the oral microbiota than asymptomatic atherosclerosis controls^12^. A previous study of patients with symptomatic atherosclerosis showed that the *Collinsella* genus was enriched in patients with symptomatic atherosclerosis, whereas *Roseburia* and *Eubacterium* were enriched in healthy controls. A previous study also suggested that the gut metagenome may be associated with the inflammatory status of hosts and patients with symptomatic atherosclerosis and may be associated with characteristic changes^13^.

The gut microbiota may also have an important role in the development of complex metabolic abnormalities in the host that consequently influence the progression of atherosclerotic heart disease. Recent studies have demonstrated that trimethylamine (TMA) and trimethylamine *N*-oxide (TMAO), which are metabolites derived from the gut microbiota, are associated with CVD and atherosclerosis^14,15^. Metabolism of dietary L-carnitine, a trimethylamine that is abundant in red meat, by the gut microbiota also produces TMAO and accelerates atherosclerosis in mice^16^. Bacterial dysbiosis can also influence gut immunity, which increases the risk of atherosclerotic heart disease^17^. Recent research has revealed that patients with chronic inflammatory bowel diseases, such as Crohn’s disease and ulcerative colitis, are at increased risk of acute arterial events, and young patients have the highest risk. Disease activity may have an independent impact on acute arterial events^18^. However, the composition and functional capacity of the gut microbiome in relation to CVD and atherosclerosis have not been systematically examined. Additionally, it remains unclear how the gut microbiota interacts with CVD in terms of immunity.

In this study, we examined the effects of the gut microbiota on the pathogenesis of atherosclerosis in a transgenic atherosclerosis model with C1q/TNF-related protein 9-knockout (CTRP9-KO) mice. CTRP is an adipokine and an adiponectin family paralog. Members of the CTRP family directly or indirectly play protective roles in regulating the inflammatory cascade and the immune and cardiovascular systems^19–22^. CTRP9, which is a member of the CTRP family, plays an important role in cardiovascular homeostasis^23,24^, promotes endothelial cell function, and improves endothelial-dependent vasorelaxation^25^.

CTRP9-KO mice showed altered gut microbial composition and significantly increased cholesterol and LDL levels compared to wild-type control mice. FMT experiments showed that gut microbial composition was significantly associated with the progression of atherosclerosis. The transplantation of WT stools into KO mice protected against the progression of atherosclerosis. The transplantation of KO stools into WT mice promoted the progression of atherosclerosis. In addition, these effects were linked to macrophages. Our results elucidate a critical role of the gut microbiota in atherosclerosis pathogenesis. In other words, genetic variations that affect atherosclerosis alter the composition of the gut microbiome. In addition, altered gut microbial composition affects the progression of atherosclerosis, suggesting that fecal microbiota transplantation may help to prevent atherosclerosis.

## Materials and methods

### Experimental animals

All mice were housed in ambient RT (22 ± 1 °C) with 12/12 h light-dark cycles and free access to water and food. Ctrp9-knockout (KO) mice were generated using conventional CRISPR/Cas9 technology, as previously described^26^. Ctrp9-specific guide RNA and Cas9 mRNA were microinjected into the cytoplasm of C57BL/6 N mouse zygotes. The surviving embryos were transferred into the oviducts of synchronized pseudopregnant foster mothers. Genomic DNA samples were prepared from tail biopsies to screen for founder mice with potential targeted mutations in Ctrp9. The selected mutant allele was a 22 bp deletion in Ctrp9 that induced a frame shift followed by a premature stop codon (Supplementary Fig. 1a–d).

To maintain the same conditions for all animal experiments other than CTRP9 gene deletion, in this study, all animals used were the genetically identified C57BL/6 N strain. All mice used in our experiments were male. SPF C57BL/6 J mice (3–8 weeks old) were obtained from Orient Bio in Seoul, Korea. All mice were raised under controlled appropriate SPF conditions with a temperature of 24 ± 1 °C, humidity of 50% to 70% and 12-h light-dark cycles, with the lights on from 8:00 a.m. to 8:00 p.m. The animals were housed in individual sterile plastic cages (exhaust ventilated closed-system cage rack). A standard sterile diet (Purina 5057) was purchased and fed to the mice. Drinking water was sterilized through the 1st microfilter, 2nd carbon filter, 3rd RO water (reverse osmosis) and 4th UV sterilization device.

### DNA extraction and bacterial metagenomic analysis

Fecal metagenomic DNA was extracted using a DNA isolation kit (PowerSoil DNA Isolation Kit, MO BIO, USA). Bacterial DNA was quantified using the QIAxpert system (QIAGEN, Germany). The 16 S rRNA gene targeting V3–V4 of the variable region was amplified with 16S_V3_F (5′- TCGTCGGCAGCGTCAGATGTGTATAAGAGACAGCCTACGGGNGGCWGCAG-3′) and 16S_V4_R (5′- GTCTCGTGGGCTCGGAGATGTGTATAAGAGACAGGACTACHVGGGTATCTAATCC-3′) primers. Libraries were prepared using PCR products according to the MiSeq System guide (Illumina, USA) and quantified using QIAxpert (QIAGEN, Germany). Each amplicon was then quantified, set at an equimolar ratio, pooled, and sequenced on a MiSeq instrument (Illumina, USA) according to the manufacturer’s recommendations.

Paired-end reads matching the adapter sequences were trimmed by Cutadapt version 1.1.6. The resulting FASTQ files containing paired-end reads were merged with CASPER and then quality filtered with the Phred (Q) score-based criteria described by Bokulich^27,28^. Low-quality reads shorter than 350 bp and longer than 550 bp after being merged were also removed. To identify chimeric sequences, a reference-based chimera detection step was conducted with VSEARCH against the SILVA gold database^29,30^. Sequence reads were then clustered into operational taxonomic units (OTUs) using VSEARCH with a de novo clustering algorithm and a threshold of 97% sequence similarity. The compositions and proportions of bacterial species were finally classified using the SILVA 128 database with UCLUST (parallel_assign_taxonomy_uclust.py script on QIIME version 1.9.1) and default parameters^31^. The Chao index, an estimator of the richness of taxa per individual, was calculated to measure the diversity of each sample.

### Cross-transplantation of fecal microbiota

Fecal microbiota transfer was performed as described by Ericsson et al. ^32^. Fresh fecal samples were collected from 6-week-old C57BL/6 WT mice and CTRP9-KO mice and stored at −80 °C. All experimental mice were treated with antibiotics (1 g/L ampicillin, 0.5 g/L vancomycin, 1 g/L neomycin, and 1 g/L metronidazole) in their drinking water for 3 weeks. The fecal matter was suspended in PBS and passed through a 30 μm pore nylon filter to remove large particulate and fibrous matter. Fresh fecal slurries were administered intragastrically to recipient mice. FMT was initiated in mice at 9 weeks of age after 3 weeks of antibiotic treatment and was carried out twice per week for a total of 3 weeks. WT mice were inoculated with fecal material derived from CTRP9-KO mice, and CTRP9-KO mice were inoculated with fecal material from WT mice. WT and CTRP9-KO mice that were not inoculated were used as controls.

### Atherogenic animal models

To assess atherosclerotic pathogenesis, partial carotid ligation was performed as previously described with minor modifications^33^. Briefly, 8-week-old male mice were anesthetized by 2.5% isoflurane inhalation (Piramal Critical Care, Inc. Bethlehem, PA, USA). Then, the left external and internal carotid arterial branches were ligated with 6-0 silk sutures. After ligation, the mice were fed a high-fat diet (HFD; D12336, Research Diets, Inc., NJ, USA) to maximize atherosclerosis development. Three weeks later, the left carotid artery, right carotid artery, whole aorta, and blood samples were collected to verify atherosclerotic development.

### Biological analysis of blood samples

Blood samples were obtained from the vena cava before sacrifice and centrifuged at 3500 × *g* for 10 min at 4 °C. Total cholesterol (TC), triglyceride (TG), high-density lipoprotein (HDL), and low-density lipoprotein (LDL) were measured by a blood biochemical analyzer (Hitachi 7180, Hitachi, Tokyo, Japan).

### Tissue preparation and histologic analysis

To study arterial morphology, vessels were perfused with PBS. Whole aortas and carotid arteries were rapidly removed, washed in PBS and fixed with 4% paraformaldehyde. Aortas were stained with oil red O (ORO) as described. ORO powder was suspended in propylene glycol at a final concentration of 0.4%. After the fixed aortas were symmetrically cut, they were washed with serially diluted propylene glycol (60–90%) and stained with 0.4% ORO. Fixed carotid artery samples were embedded in paraffin, sectioned at a thickness of 4 µm, and stained with hematoxylin and eosin (H&E).

### Immunofluorescence staining

Immunofluorescence staining was performed using α-SMA (ab5694, Abcam, Cambridge, MA, USA), CD31 (Abcam, Cambridge, MA, USA), CD45 (550539, BD Pharm., San Jose, CA, USA), CD3 (A0452, DAKO, Santa Clara, CA, USA), CD19 (14-0194-82, Thermo, Waltham, MA, USA), CD68 (ab125212, Abcam, Cambridge, MA, USA), and Sca-1 (AF1226, R&D Systems, Minneapolis, MN, USA) primary antibodies. Anti-rabbit AF546, anti-mouse AF488, and anti-rat AF594 secondary antibodies were used, and DAPI (Molecular Probes, Eugene, OR, USA) was used to stain the nuclei. Mounted tissue slides were photographed using an LSM 780 laser scanning microscope (Carl Zeiss, Germany). Intensity profiles were analyzed using ZEN 2.6 (blue edition) software.

### Statistical analysis

All data are presented as the mean ± SD of at least three independent experiments for a given sample. Two-tailed Student’s t test was used for comparisons between two groups. *P* values < 0.05 were deemed significant.

## Results

### Construction of atherosclerosis-prone CTRP9-KO mice

**Fig. 1:**
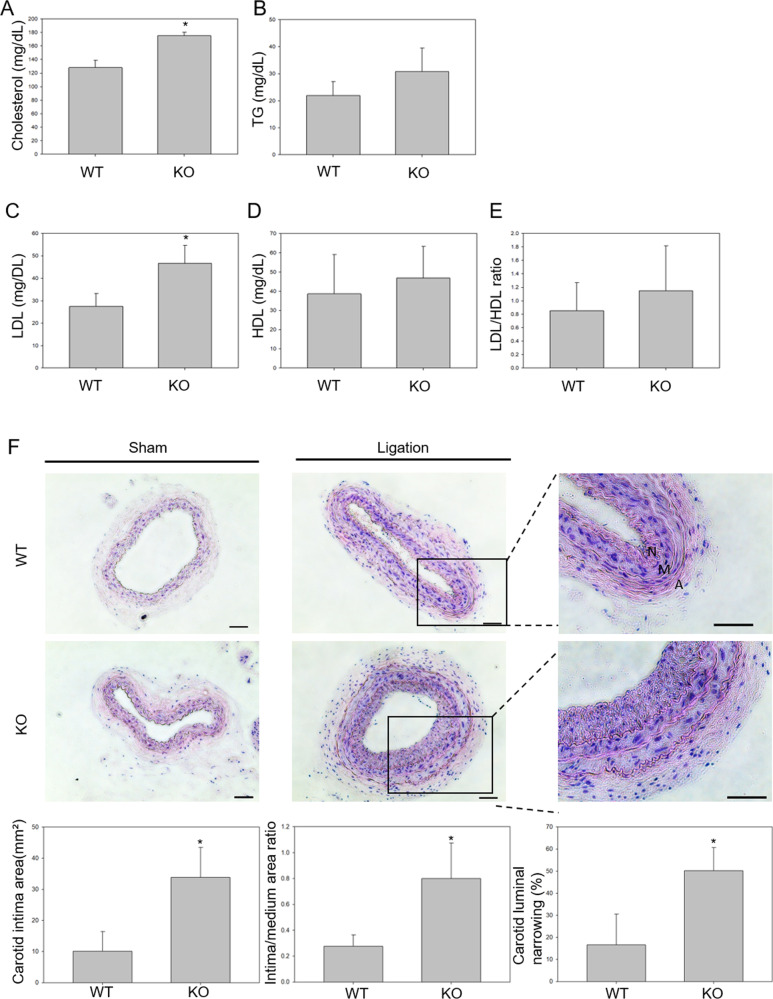
Construction of atherosclerosis-prone CTRP9-KO mice. **a**–**e** Serum profiles of cholesterol, TG, LDL, HDL, and the LDL/HDL ratio in WT and CTRP9-KO mice. **f** Representative micrographs of H&E-stained carotid artery lesions 3 weeks after partial ligation and HFD feeding. Scale bars in right lower corner represent 50 μm.

To determine the functional consequences of CTRP9 gene disruption, a partial carotid ligation model was used to evaluate the effects on atherosclerosis progression, and the mice were fed a HFD for three weeks. HFD-fed CTRP9-KO mice had elevated levels of serum cholesterol and low-density lipoprotein compared to WT mice (Fig. 1a–c), while TG and high-density lipoprotein levels were not significantly different (Fig. 1d, e). To identify more evidence of the atherogenic effects of CTRP9 deficiency, histologic analysis of the left carotid artery (LCA) was performed. At 3 weeks after LCA partial ligation and HFD feeding, neointima formation and luminal narrowing were significantly induced in CTRP9-KO mice (Fig. 1f). Collectively, these results indicate that CTRP9 plays a crucial role in atherosclerosis progression.

### Alterations in the gut microbial community resulting from CTRP9 gene deficiency

**Fig. 2:**
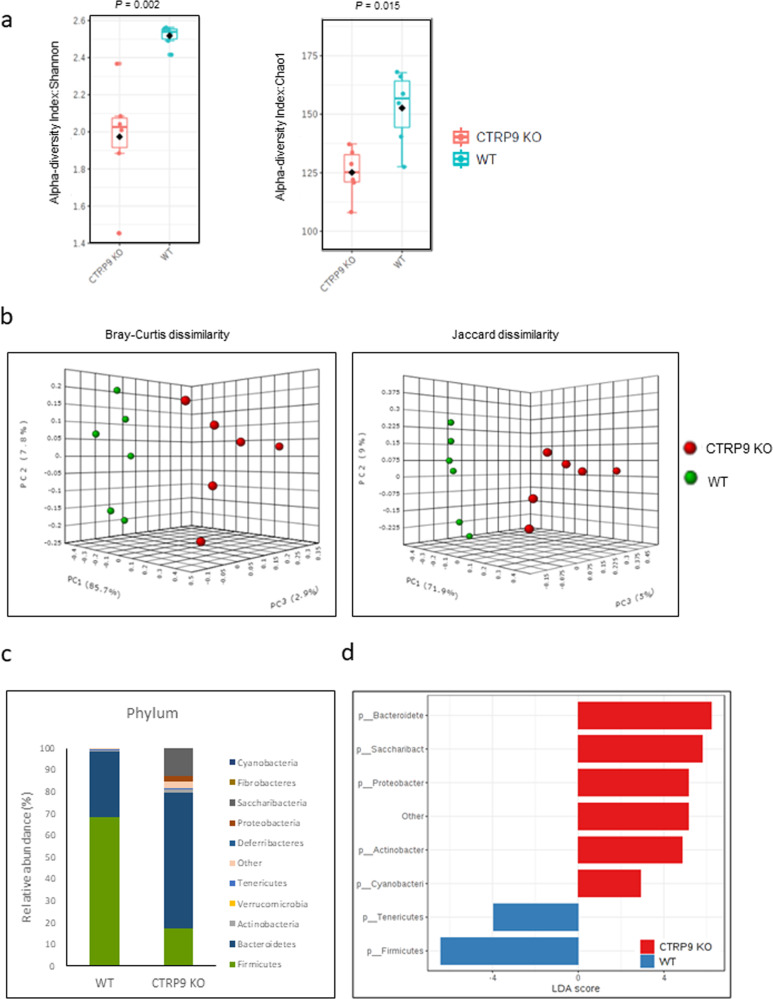
Alterations in gut microbiota composition in CTRP9-KO mice with atherosclerosis. The V3-V4 region of bacterial 16 S rRNA was sequenced in fecal samples from 6-week-old CTRP9-KO (*n* = 6) and WT (*n* = 6) mice. **a** Alpha diversity analysis based on Shannon’s index (proxy for diversity) and Chao1 (proxy for richness) at the phylum level. **b** Beta-diversity analysis based on Bray–Curtis (qualitative measure) and Jaccard distances (quantitative measure). **c** Relative abundance of the total gut microbiota at the phylum level in CTRP9-KO and WT mice. **d** Percentage of bacterial taxa discriminating the gut microbiota in CTRP9-KO and WT mice.

To explore whether the development of atherosclerosis was associated with intestinal dysbiosis, the gut metagenomic profile of atherosclerosis-prone CTRP9-KO mice was compared to that of WT mice at 6 weeks. To assess how each genotype affects the gut microbial profile, alpha- and beta-diversity were compared to assess microbial diversity in these two groups. Alpha diversity was analyzed by calculating Shannon’s index (a proxy for diversity) and Chao1 (a proxy for richness). There were significant differences in bacterial diversity and richness between CTRP9-KO and WT mice (Fig. 2a). Shannon’s diversity, which is an indirect measure of evenness, was significantly lower in the CTRP9-KO group than in the WT group (Kruskal–Wallis test, *p* = 0.002). Chao1 richness was significantly higher in WT mice than in CTRP9 mice (Kruskal–Wallis test, *p* = 0.015). Next, the beta diversity in the two groups was analyzed using Bray–Curtis (qualitative measure) and Jaccard distances (quantitative measure) (Fig. 2b). The gut microbiota of CTRP9-KO mice was different from that of WT mice in terms of qualitative (*P* < 0.001) and quantitative measures (*P* < 0.001) of bacterial clustering, indicating that CTRP9-KO mice have an altered gut microbial community. These observations suggest that alterations in the gut microbiota could be attributed to changes in the CTRP9 gene.

Next, the percentage of bacterial taxa differentiating the gut microbiotas of CTRP9-KO and WT mice was calculated using a linear discriminant analysis effect size (LEfSe) method (Fig. 2c, d). Different profiles were noted for CTRP9-KO and WT mice. The most abundant phylum was *Firmicutes* (68.38%; 95% CI, 62.47–73.73%) in WT mice, but *Bacteroidetes* (62.38%; 95% CI, 50.82–74.53%) was the most abundant phylum in CTRP9-KO mice (Fig. 2c). The taxonomy of the phyla of gut microbiota in CTRP9-KO and WT mice is summarized in Supplementary Table 1. The bacterial diversity of WT mice was slightly higher than that of CTRP9-KO mice. The *Ruminococcaceae* family of phylum *Firmicutes* was the most frequent (45.64%) among the gut microbiota of WT mice, and the *Bacteroidaceae* family of phylum *Bacteroidetes* was 28.99%. In CTRP9-KO mice, the *Bacteroidales* family S24-7 group of phylum *Bacteroidetes* was the most frequent (51.15%), followed by an unknown (12.60%) family from phylum *Saccharibacteria* (Table 1). The dominant gut microbiota were identified in WT and CTRP9-KO mice.

**Table 1:** Distinct gut microbiota compositions in CTRP9-KO and WT mice.

Phylum	Family					Genus				
	Taxon	Abundance (%)		*P* values	LDA score	Taxon	Abundance (%)		*p* value	LDA score
		WT	CTRP9-KO				WT	CTRP9-KO		
Firmicutes	f_Ruminococcaceae	45.64	3.89	0.004	−6.32	g_Ruminococcaceae UCG-014	28.68	1.83	0.002	−6.13
						g_Ruminococcaceae UCG-013	8.46	0.16	0.002	−5.62
						g_Ruminococcus 1	2.34	0.02	0.002	−5.07
						g_uncultured	2.15	0.19	0.002	−4.93
						g_Ruminococcaceae UCG-003	0.52	0.05	0.002	−4.37
						g_Ruminococcaceae NK4A214 group	0.18	0.05	0.009	−3.83
						g_Ruminiclostridium_5	0.31	0.12	0.026	−3.98
						g_Ruminiclostridium_6	0.17	0.004	0.044	−3.93
						g_Ruminiclostridium	0.20	0.07	0.041	−3.82
	f_Peptococcaceae	0.75	0.04	0.004	−4.54	g_uncultured	0.73	0.04	0.002	
Bacteroidetes	f_Bacteroidaceae	28.99	1.05	0.004	−6.15	g_Bacteroides	28.99	1.05	0.002	−6.15
	f_Porphyromonadaceae	0.77	0.07	0.004	−4.56	g_Parabacteroides	0.76	0.05	0.002	−4.56
	f_Bacteroidales S24-7 group	0.10	51.15	0.004	6.41	g_uncultured bacterium	0.10	50.52	0.002	6.40
						g_mouse gut metagenome	0	0.46	0.003	4.36
						g_uncultured Bacteroidales bacterium	0	0.16	0.003	3.90
	f_Prevotellaceae	0	5.76	0.002	5.46	g_Prevotella 9	0	4.48	0.003	5.35
						g_Prevotellaceae UCG-001	0	1.28	0.003	4.81
	f_Rikenellaceae	0.001	4.35	0.003	5.34	g_Rikenella	0	1.12	0.003	4.75
						g_Alistipes	0.001	3.24	0.004	5.21
Proteobacteria	f_Alcaligenaceae	0.001	0.94	0.003	4.68	g_Parasutterella	0.001	0.94	0.004	4.68
	f_Desulfovibrionaceae	0	1.85	0.002	4.97	g_Desulfovibrio	0	1.81	0.003	4.96
						g_Bilophila	0	0.04	0.003	3.25
Saccharibacteria	f_Unknown_Family	0.004	12.60	0.004	5.80	g_Candidatus Saccharimonas	0.004	12.60	0.005	5.80
Verrucomicrobia	f_Verrucomicrobiaceae	0.39	0.001	0.146	−4.29	g_Akkermansia	0.39	0.001	0.146	−4.29

Analyses were performed on WT (*n* = 6) and CTRP9-KO (*n* = 6) mice.

The data are presented as the mean percentages and LDA scores.

A nonparametric Wilcoxon test was used to compare two independent groups.

### FMT altered the composition of the gut microflora in CTRP9-KO and WT mice

**Fig. 3:**
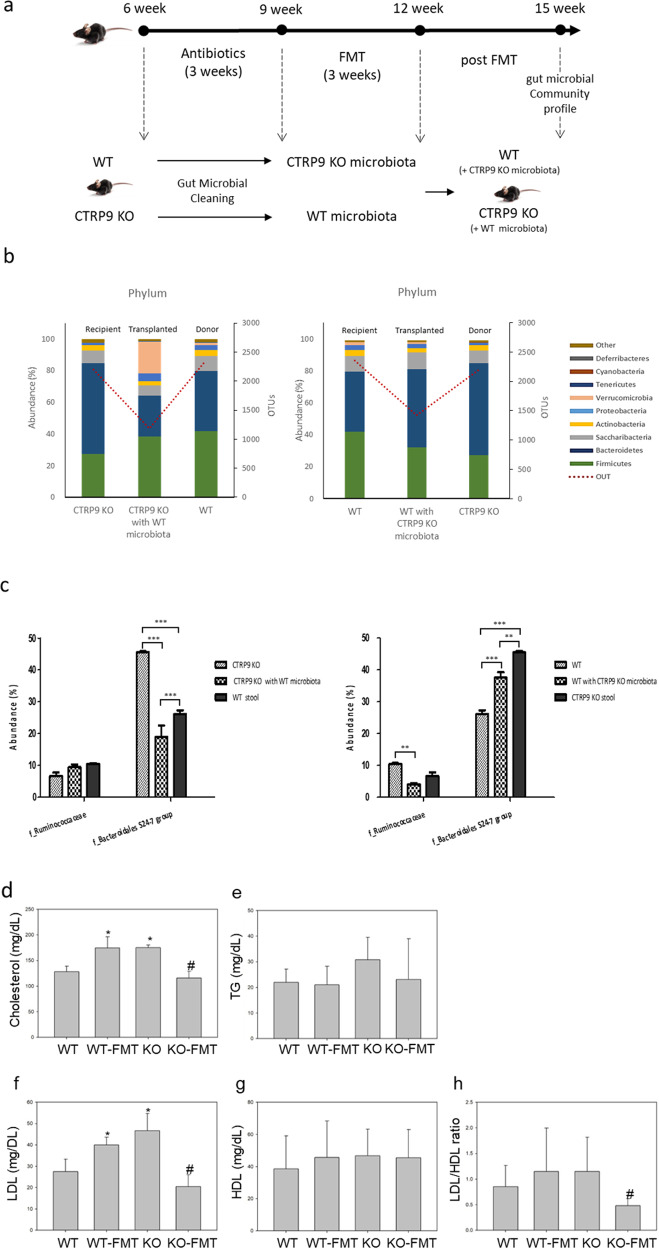
Alterations in gut microflora composition in CTRP9-KO and WT mice after FMT. **a** Study design. Animals were treated with antibiotics for 6 weeks after birth. Then, FMT was performed in the two groups (*n* = 3 per group). The WT group received fecal extracts from CTRP9-KO mice with atherosclerosis, and the CTRP9-KO group received fecal extracts from healthy WT mice. Three weeks after FMT, gut microbial community analysis and atherosclerosis analysis were performed in both groups. **b** Relative abundance of the total gut microbiota at the phylum level in both CTRP9-KO mice transplanted with WT gut microbiota and WT mice transplanted with CTRP9-KO gut microbiota. **c** Average relative abundance of the predominant microbiota at the family level in both groups. Serum levels after high fat diet (HFD) feeding for 3 weeks were measured in the WT, CTRP9-KO, WT transplanted with CTRP9-KO gut microbiota, and CTRP9-KO transplanted with WT gut microbiota groups. **d** Total cholesterol. **e** Triglyceride (TG). **f** Low-density lipoprotein (LDL). **g** High-density lipoprotein (HDL). **h** The LDL/HDL ratio. The data are expressed as the mean ± standard error of the mean (*<0.05 using Student’s *t* test *n* = 3/group).

To clarify the association between alterations in the gut microbiota and the pathogenesis of atherosclerosis, FMT was performed in the two groups of mice (Fig. 3a). First, WT mice were used as donors, and CTRP9-KO mice were used as recipients. Next, the fecal microbiota of CTRP9-KO mice was transplanted to WT mouse recipients. FMT effectiveness was evaluated by comparing the gut microbiota profiles of recipient, transplanted, and donor mice. FMT largely affected the gut microbiota of CTRP9-KO and WT mice, and all transplanted mice acquired the gut microbiota of donor mice (Fig. 3b). The composition of the gut microbiota in FMT mice was significantly changed after 3 weeks of FMT (*P* < 0.05). CTRP9-KO mice that were transplanted with the gut microbiota of WT mice showed a significant increase in *Firmicutes* from 27.13% to 38.42% compared to that in recipient mice, whereas *Bacteroidetes* decreased from 57.63% to 25.73%. In WT mice transplanted with the CTRP9-KO gut microbiota, *Bacteroidetes* increased from 37.88% to 48.91% and *Firmicutes* decreased from 47.70% to 32.11%. The most frequent family in WT mice, *Ruminococcaceae*, increased from 6.67% to 9.46% in CTRP9-KO mice after the mice were transplanted with the WT gut microbiota, while the most frequent family in CTRP9-KO mice, *Bacteroidales* S24-7 group, decreased significantly from 45.57% to 18.97%. In WT mice transplanted with the CTRP9-KO gut microbiota, the f_Bacteroidales S24-7 group increased from 26.19% to 37.62% (*p* < 0.001), while f_Ruminococcaceae decreased significantly from 10.53% to 4.10% (*p* < 0.001) (Fig. 3c).

### Transplantation with WT gut microbiota ameliorated atherosclerosis in CTRP9-KO mice

Based on these results, we verified the effectiveness of FMT administered by oral injection. To examine the effects of FMT on atherosclerosis progression in detail, a partial carotid ligation model was used to evaluate the transplantation of the gut microbiota in vivo, as described in the Methods. Serum lipid levels in WT and CTRP9-KO mice were the same as those shown in Fig. 1a–e. In WT mice that were transplanted with the microbiota from CTRP9-KO mice, cholesterol and LDL levels were noticeably increased (Fig. 3d, f, and h), similar to those of CTRP9-KO mice, but TG and HDL levels were not significantly altered (Fig. 3e, g). In CTRP9-KO mice that were transplanted with the microbiota of WT mice, cholesterol and LDL levels were substantially decreased, while TG levels were decreased more than those in the WT group (Fig. 3d, f).

**Fig. 4:**
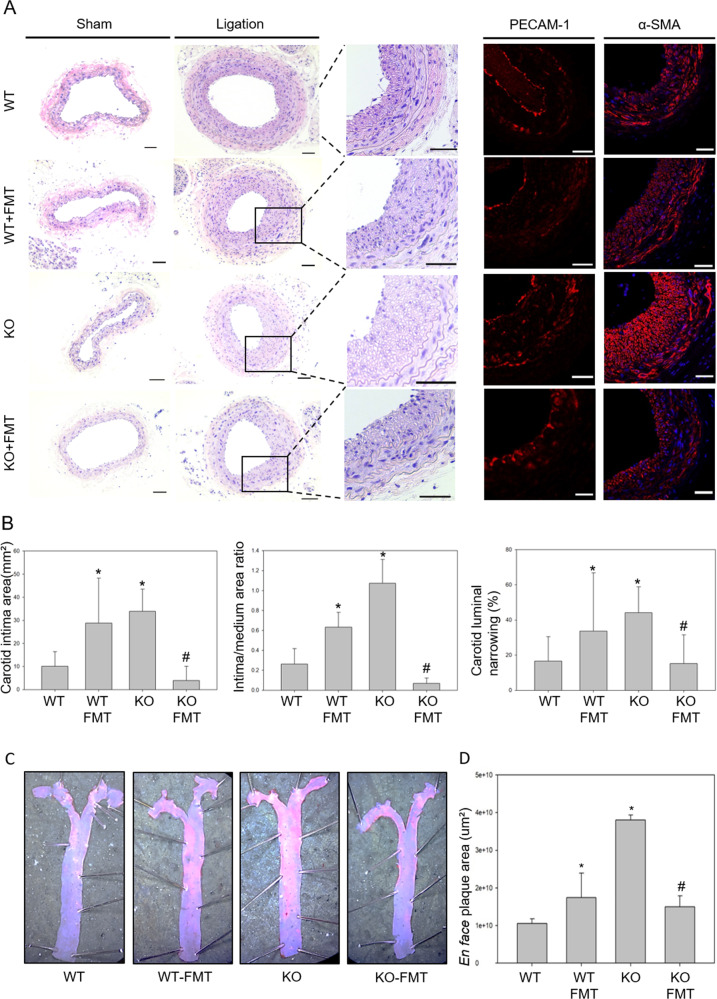
Amelioration of atherosclerosis in CTRP9-KO mice transplanted with the WT gut microbiota. **a** Representative micrographs of H&E-stained carotid lesions 3 weeks after partial ligation and HFD feeding. Sham, sham surgery group; ligation, ligation surgery group. Squares indicate areas shown at higher-power magnification of the respective cross-sections. Representative images showing immunofluorescence staining of PECAM-1 and α-SMA in carotid lesions. **b** Micrographs were quantified using ImageJ software. **c** Atherosclerotic plaques in the aorta were stained en face with oil red O. Representative en face view of the aortic surface area covered by lesions after 3 weeks of partial ligation and HFD feeding. **d** Micrographs were quantified using ImageJ software. Error bar indicates the SD (*n* = 3). The results show the mean ± SD. *p < 0.05 for the WT-FMT group and KO group compared to the WT group. #*p* < 0.05 for the KO-FMT group compared to the KO group. All group comparisons were performed with *t* test. Scale bars in right lower corner represent 50 μm.

Histologic analysis was used to further examine the effect of microbiota transplantation on atherosclerosis progression. The carotid arteries were isolated, and a paraffin block was produced. H&E staining of carotid artery sections was used to measure neointima formation and luminal narrowing (Fig. 4a, b). As expected, CTRP9-KO mice had significantly more atherosclerotic lesions than WT mice. Atherosclerotic lesions in the carotid arteries were increased in WT mice with FMT more than in WT mice without FMT, while lesions were decreased in CTRP9-KO mice with FMT more than in CTRP9-KO mice without FMT. Moreover, CTRP9-KO mice with FMT showed decreased carotid luminal narrowing compared to WT mice (Fig. 4b). Immunofluorescence staining of CD31 and SMA showed the same results: significant proliferation in the media and neointima in WT mice with FMT and CTRP9-KO mice without FMT but not in WT mice without FMT or CTRP9-KO mice with FMT (Fig. 4a). To obtain additional evidence of effect of fecal microbiota transfer on atherosclerotic lesions in WT and CTRP9-KO mice, whole aortas from HFD-fed WT and CTRP9-KO mice with or without FMT were stained en face with oil red O (ORO). Quantitative analysis revealed statistically significant numbers of lesions. Atherosclerotic lesions were increased in WT mice with FMT and decreased in CTRP9-KO mice with FMT (Fig. 4c, d). To summarize the results thus far, the data showed the same pattern in which CTRP9-KO mice had increased atherosclerosis progression compared to WT mice, while microbiota transfer resulted in the opposite effects. Symptoms were exacerbated by treatment with KO stool, while treatment with WT stool relieved symptoms. These interesting results indicate that CTRP9-KO mice are an effective animal model for atherosclerosis studies, and the transfer of fecal microbiota between WT mice and CTRP9-KO mice showed a useful therapeutic effect on atherosclerosis.

### The vasa vasorum plays a crucial role in the accumulation of immune cells and atherosclerosis progression in CTRP9-deficient mice

Thus far, we have shown that the adipokine CTRP9 plays an important role in atherosclerosis progression and that FMT can protect against the progression of atherosclerosis. Next, we further examined angiogenesis in the affected adventitial vasa vasorum and the distribution of the immune cell populations in atherosclerotic lesions after fecal microbiota transfer between CTRP9-KO and WT mice.

**Fig. 5:**
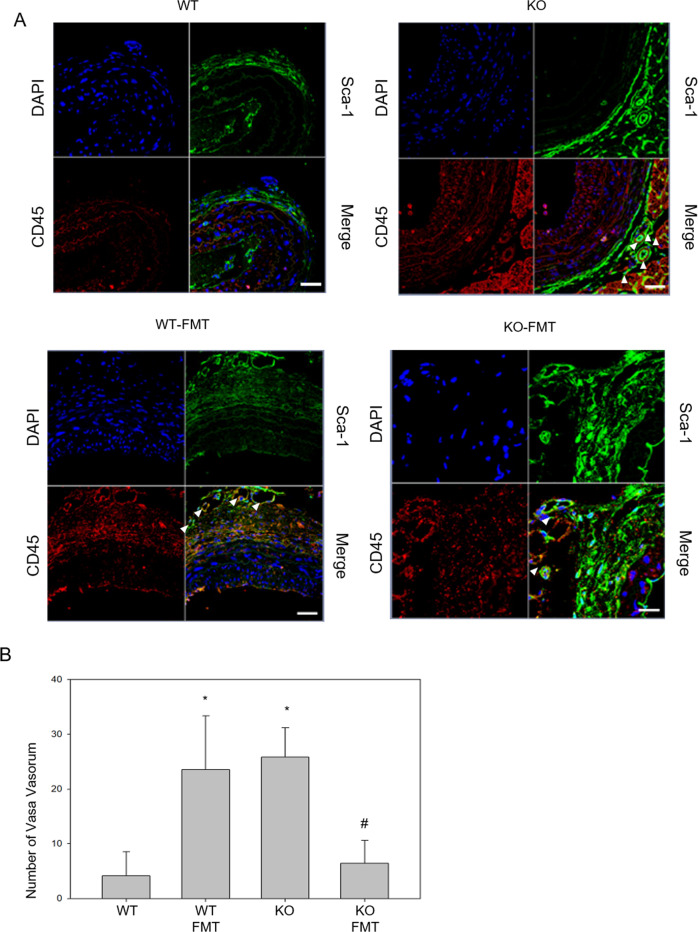
Adventitial vasa vasorum contains CD45 + Sca-1 + cells in the atherosclerotic aorta. **a**, **b** Vasa vasorum staining with CD45 and Sca-1. White arrows indicate the vasa vasorum, #*p* < 0.05 the KO-FMT group compared to the KO group. Two-way comparisons were performed with t tests. Scale bars in right lower corner represent 50 μm.

The adventitial vasa vasorum was identified as CD45 + Sca-1+ cells in the atherosclerotic aorta (Fig. 5a, b). The vasa vasorum plays a role in blood supply and nourishment for the tunica adventitia and outer parts of the tunica media in large veins and arteries. A previous report showed that CD45 + Sca-1+ cells are vasculogenic and that the vasa vasorum plays a role in the development of atherosclerosis^34^. Double staining with CD45 and Sca-1 was performed. Cells forming the vessel were completely stained with CD45 and Sca-1 to identify the vasa vasorum. The number of vasa vasorum vessels was significantly increased in CTRP9-KO mice without FMT and WT mice with FMT compared to WT mice without FMT and CTRP9-KO mice with FMT (Fig. 5a, b). These findings suggest that vasa vasorum pathology plays an important role in atherosclerosis progression due to CTRP9 deficiency. In addition, the exchange of the gut microbiota from WT mice by FMT restored the function of the vasa vasorum.

**Fig. 6:**
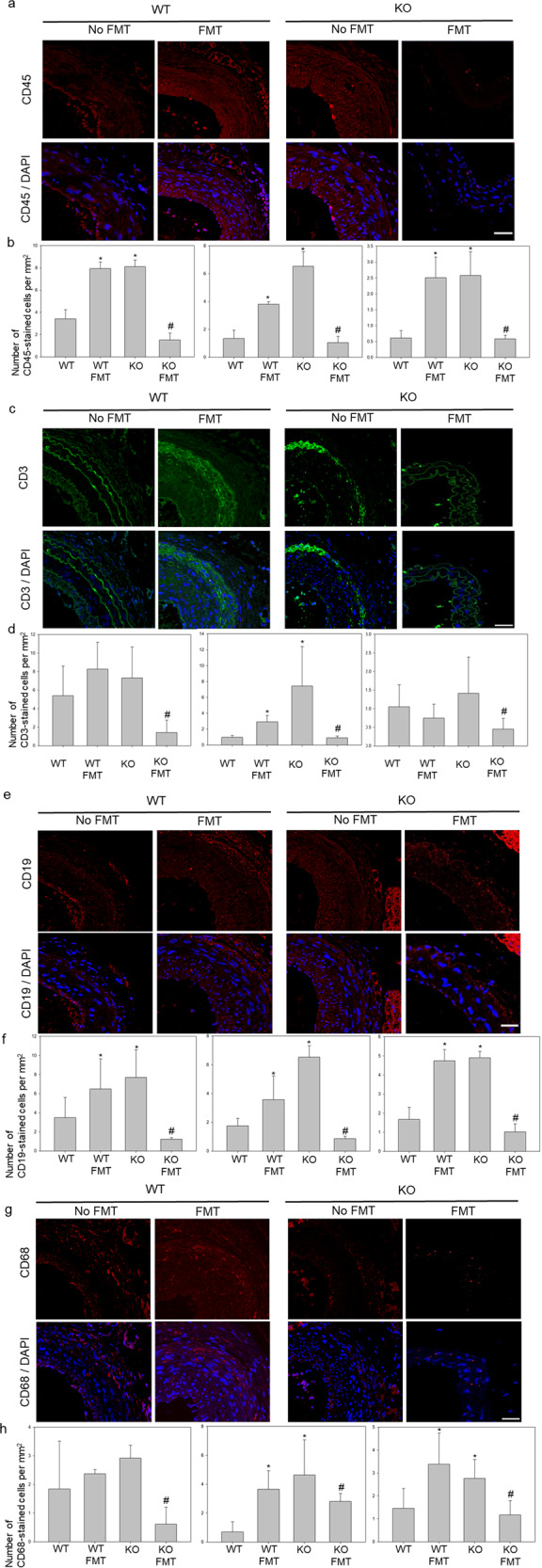
The accumulation of immune cells results in atherosclerosis progression. **a**, **b** Immunofluorescence staining for the lymphocyte marker CD45 (red) and DAPI-stained nuclei (blue) are shown. **c**, **d** T cells were stained for CD3. **e**, **f** B cells were stained for CD19. **g**, **h** Macrophages were stained for CD68. *p < 0.05 for the WT-FMT group and the KO group compared to the WT group, #*p* < 0.05 for the KO-FMT group compared to the KO group. Two-way comparisons were performed with *t* tests. Scale bars in right lower corner represent 50 μm.

As expected, the overall expression of immune cell markers was increased in CTRP9-KO mice and WT mice with FMT. First, a substantial number of CD45-stained cells was detected in the overall lesions of CTRP9-KO mice (Fig. 6a, b). WT mice with FMT had increased numbers of CD45-stained cells compared to WT mice, while CTRP9-KO mice with FMT had decreased numbers of CD45-stained cells compared to CTRP9-KO mice and WT mice. These results indicate that atherosclerosis due to CTRP9 deficiency causes the deposition of various immune cells.

Next, the populations of B cells, T cells, and macrophages were examined to identify the immune cells involved in atherosclerosis progression due to CTRP9 deficiency (Fig. 6c–h). Variations in the total numbers of macrophages and lymphocytes were significantly increased in CTRP9-KO mice and were restored by FMT. The intestinal lamina has autofluorescence that can be detected in green channels. Therefore, WT and CTRP9-KO mice with FMT were distinguished by the intestinal lamina, while CTRP9-KO and WT mice with FMT were impossible to distinguish because of broken boundaries. Interestingly, stained cells were clustered between the adventitia and media. Variations in macrophages were significantly different in each group, while T cells were altered in the media and neointima in CTRP9-KO mice with and without FMT. Macrophages were significantly increased in the adventitia, media, and neointima in CTRP9-KO mice and WT mice with FMT compared to WT mice. The score of CTRP9-KO mice more than doubled compared to that of WT mice, but that of CTRP9-KO mice with FMT decreased more than that of the other group (Fig. 6g, h). Finally, the number of macrophages was measured and varied significantly in each group. Furthermore, macrophages were significantly increased in the adventitia, media, and neointima in the KO group and in WT mice with FMT compared to WT mice. However, the number of cells per unit area was not significantly different in the neointimal region. These results indicate that CTRP9-mediated atherosclerotic lesions were associated with the distribution of immune cells resulting from adventitial vasa vasorum angiogenesis.

## Discussion

In the present study, we investigated the potential effect of the gut microbiota on atherosclerosis pathogenesis. This study reveals that alterations in the composition of the gut microbiota in CTRP9-KO mice strongly influence atherosclerosis pathogenesis. Gut-associated pathogenesis has been strongly linked to the populations of various immune cells, including macrophages, T cells, B cells, and lymphocytes.

A substantial number of studies have provided evidence for the role of the gut microbiota in human diseases, including CVD. In animal studies, successful transplantation of certain bacteria has successfully reduced atherosclerosis in mice^35,36^. The probiotic bacterium *Akkermansia muciniphila* attenuates atherosclerotic lesions by improving metabolic endotoxemia-induced inflammation by restoring the gut barrier^35^. Two other dominant species of the genus *Bacteroides* in the human gut microbiota, *Bacteroides vulgatus* and *Bacteroides dorei*, have also been shown to attenuate the formation of atherosclerotic lesions in atherosclerosis-prone mice, markedly ameliorating endotoxemia, decreasing gut microbial lipopolysaccharide production, and effectively suppressing proinflammatory immune responses^36^. *Akkermansia muciniphila*, *B. vulgatus*, and *B. dorei* did not show any differences between WT and KO mice in this study. This is probably due to differences in the relative abundances of the dominant gut microbiota in mice and humans. this result may also be due to deficiency of the CTRP9 gene.

In this study, a CTRP9-KO mouse model was used to investigate the relationship between the gut microbiome and atherosclerosis. An adiponectin family paralog has been uncovered and designated C1q/TNF-related protein (CTRP). Members of the CTRP family directly or indirectly play protective roles in regulating the inflammatory cascade and the immune and cardiovascular systems^19–22^. Compared with other family members, CTRP9 has been the subject of more published research concerning CVD. Previous studies showed genetic evidence for a physiological role of CTRP9, suggesting that CTRP9 may play a significant role in cardiovascular homeostasis^23,24^. CTRP9 also promotes endothelial cell function and improves endothelium-dependent vasorelaxation^25^. Recently, others have shown that plasma levels of CTRP9 are reduced in obese mice^25,36^.

CTRP9 exerts a protective effect by stimulating eNOS phosphorylation and nitric oxide production in endothelial cells and reduces neointimal formation in a mouse model of vascular injury^25,37,38^. The composition of the gut microbiota in CTRP9-KO mice differed from that of WT mice at 6 weeks of age in this study, suggesting that alterations in the composition of the gut microbiota in CTRP9-KO mice may be due to transgenic modifications leading to loss of the CTRP9 gene. The most abundant phylum in CTRP9-KO mice was *Firmicutes*, whereas *Bacteroidetes* was the most abundant phylum in WT mice (Fig. 2c, d). Few discriminant bacterial taxa were detected in CTRP9-KO mice (Table 1). The bacterial diversity of CTRP9-KO mice was also slightly higher than that of WT mice (Fig. 2c). These observations suggest that community-level alterations in the gut microbiota rather than changes in a single bacterial taxon may be associated with the development of atherosclerosis in CTRP9-KO mice. This change in the gut environment of CTRP9-KO mice has been shown to significantly reduce cholesterol, LDL, and TG levels. The present study used 16 S rRNA sequencing analysis and did not identify which specific species were associated with atherosclerosis due to limited discriminatory abilities for some genera.

It is worth noting that FMT-induced modifications in the gut microbiota in CTRP9-KO mice appeared to ameliorate the etiology of atherosclerosis. FMT greatly influenced the composition of the gut microbiota in CTRP9-KO mice. As shown in the gut environment of WT mice, *Firmicutes* was greatly increased and *Bacteroidetes* was greatly decreased by FMT (Fig. 3b, c). This study demonstrates that FMT is an effective way to control the gut environment. FMT is also useful in studying the association of microbial communities with atherosclerosis pathogenesis.

To confirm that the changes in the composition of immune cells in atherosclerotic lesions depend on alterations in gut microbial communities, angiogenesis in the adventitial vasa vasorum was also evaluated, as well as the distribution of immune cells in atherosclerotic lesions in CTRP9-KO and WT mice with or without FMT. The number of vasa vasorum was significantly increased in CTRP9-KO mice without FMT and WT mice with FMT compared to WT mice without FMT and CTRP9-KO mice with FMT. These findings suggest that the pathologic vasa vasorum plays an important role in atherosclerosis progression resulting from CTRP9 deficiency, and the exchange of the gut microbiota in WT mice by FMT rescued the pathologic vasa vasorum. The number of each type of immune cell during the progression of atherosclerotic lesions was increased in CTRP9-KO mice and WT mice with FMT. These results indicate that atherosclerosis due to CTRP9 deficiency causes the accumulation of various immune cells through pathologic vasa vasorum angiogenesis. Variations in the total numbers of macrophages and lymphocytes were significantly increased in CTRP9-KO mice and were restored by FMT. Macrophages are central in the development of atherosclerosis, as they absorb deposited lipoprotein and amplify local inflammation. Previous studies have shown that C1q/TNF-related proteins (CTRPs) play various roles in vascular functions, such as the inflammatory responses of macrophages^39,40^. CTRP9 exerts its biological effects through AdipoR1 and AdipoR2^41,42^. Additionally, several groups have reported the expression of the adiponectin receptors adipoR1 and AdipoR2 in the colonic epithelium^43^.

In humans, imbalances in the composition of the gut microbiome are independent risk factors for atherosclerosis, but microbiome-environment interactions and the pathogenetic mechanisms involved remain unclear. Further studies are needed to evaluate the use of immune-targeted therapies for atherosclerotic cardiovascular disease.

In this study, we showed that mutations in the genetic background can alter the composition of the gut microbiome and result in atherosclerosis. In addition, FMT using healthy donor stool can protect against this disease in CTRP9-deficient mice. Our results indicate the possibility of controlling gut microbial composition to treat arteriosclerosis caused by genetic deficiency. FMT may be an attractive therapeutic strategy for atherosclerosis.

## Supplementary information

supplemental material
