# Supplementary material for: Fecal microbiota transplantation ameliorates atherosclerosis in mice with C1q/TNF-related protein 9 genetic deficiency
Source: Exp Mol Med. 2022 Feb 3;54(2):103–14. doi: 10.1038/s12276-022-00728-w (PMC8894390; doi:10.1038/s12276-022-00728-w)
Supplement: Supplementary file 1 — supplemental material [file 12276_2022_728_MOESM1_ESM.pdf]

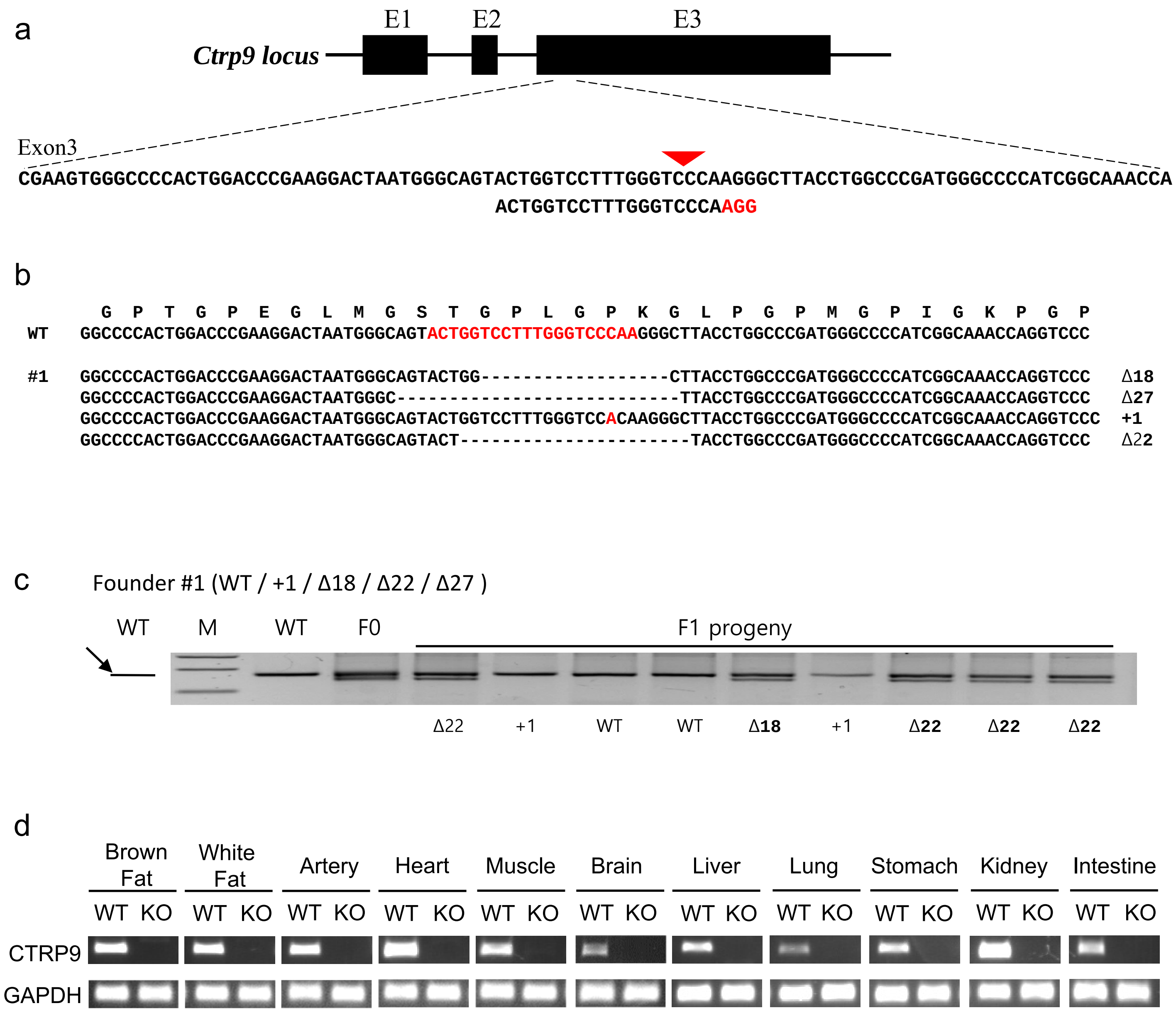

Generation of Ctrp9 knockout mice using CRISPR/Cas9. (a) The target region of validated Ctrp9 CRISPR/Cas9 in the mouse Ctrp9 locus. PAM is shown in red and target is denoted by black characters. (b) DNA sequences of the Ctrp9 locus from live founder (F0) mouse (#1) identified by sequencing analysis. '-' denotes deleted nucleotides. (c) Germ-line transmission of CRISPR/Cas9 induced Ctrp9 mutant alleles. Selected F0 mouse was crossed to wild-type mice, and the genotypes of their F1 progeny were determined by PCR genotyping. WT, wild-type; F0, F0 founder; M, marker DNA. (d) Agarose gel electrophoresis based genotyping assays identifying knockout mice derived from vital organs mRNA.

Taxonomy summary of gut microbiota phyla in CTRP9 KO and WT mice.

Taxon	Abundance (%)		*P*-value
	WT	CTRP9 KO	
Firmicutes	68.38	17.06	**0.002**
Bacteroidetes	29.86	62.38	**0.002**
Actinobacteria	0.544	1.90	**0.015**
Verrucomicrobia	0.392	0.001	0.146
Tenericutes	0.340	0.151	**0.041**
Deferribacteres	0.131	0.026	0.173
Proteobacteria	0.025	2.86	**0.002**
Saccharibacteria	0.004	12.60	**0.005**
Fibrobacteres	0.001	0.000	0.176
Cyanobacteria	0	0.016	**0.003**
Other	0.319	3.01	**0.002**

Analysis was performed with WT (n=6) and CTRP9 KO (n=6) mice.

Data are presented as mean percentages.

Non-parametric Wilcoxon test was applied to compare two independent groups.
